# 207. The increased frequency and clinical characteristics of *Streptococcus agalactiae* serotype VIII from blood isolates at two tertiary care hospital in South Korea over 14 years

**DOI:** 10.1093/ofid/ofad500.280

**Published:** 2023-11-27

**Authors:** Min Ji kim, Sehyun Ji, Ahrang Lee, Sarah Kim, Yoon Jung Lee, Hae Sung Jeong, Sung un Shin, Uh Jin Kim, Seung-Ji Kang, Kyung-Hwa Park, Sook-In Jung, Seong Eun Kim

**Affiliations:** Department of Infectious Diseases, Chonnam National University Medical School,Gwangju, Korea., Gwangju, Kwangju-jikhalsi, Republic of Korea; Department of Infectious Diseases, Chonnam National University Medical School,Gwangju, Korea., Gwangju, Kwangju-jikhalsi, Republic of Korea; Department of Infectious Diseases, Chonnam National University Medical School,Gwangju, Korea., Gwangju, Kwangju-jikhalsi, Republic of Korea; Chonnam National University Hospital, GwangJu, Kwangju-jikhalsi, Republic of Korea; Department of Infectious Diseases, Chonnam National University Medical School,Gwangju, Korea., Gwangju, Kwangju-jikhalsi, Republic of Korea; Department of Infectious Diseases, Chonnam National University Medical School,Gwangju, Korea., Gwangju, Kwangju-jikhalsi, Republic of Korea; Chonnam National University hospital, Kangju, Kwangju-jikhalsi, Republic of Korea; Chonnam National University Medical School, GwangJu, Kwangju-jikhalsi, Republic of Korea; Infectious Diseases, Gwang-ju, Kwangju-jikhalsi, Republic of Korea; Chonnam National University Medical School, GwangJu, Kwangju-jikhalsi, Republic of Korea; Department of Infectious Diseases, Chonnam National University Medical School,Gwangju, Korea., Gwangju, Kwangju-jikhalsi, Republic of Korea; Chonnam National University Medical School, GwangJu, Kwangju-jikhalsi, Republic of Korea

## Abstract

**Background:**

Invasive group B *Streptococcus* (GBS) infections are increasing among non-pregnant adults with underlying diseases. Previous studies have shown that the proportion of GBS serotype of pregnant women were differed from geographic regions. However, studies on the serotype of invasive GBS infection in non-pregnant adults are sparse. This study aimed to evaluate epidemiology and clinical characteristics of strains caused GBS bloodstream infection according to serotypes.

**Methods:**

*S. agalactiae* blood isolates were collected between March 2007 and December 2021, and their clinical data were obtained from two tertiary hospital of South Korea. Species identification was performed using MALDI-TOF MS and serotyping test was performed using commercial latex particle agglutination kit. Antimicrobial susceptibility tests are conducted using automated equipment.

**Results:**

A total of 130 patients with GBS bacteremia were identified and among them 125 isolates were serotypeable. The median age were 66 years and 46% were male. Serotype VIII was most dominant (31.5%) and Ib, III, Ia, V were as follows (20.8%, 13.8%, 12.3%, 11.5% retrospectively). The proportion of serotype VIII increased from 27.3% to 53.3% from 2015 to 2021. Most common source of GBS bloodstream infection were primary bacteremia (34.4%), followed by bone and joint infections (16.8%) and skin and soft tissue infections (13.6%). Cardiovascular disease (79.2%) was most frequently reported in GBS bacteremia patients, followed by malignancy (44.6%) and diabetes mellitus (40%). The 30-day mortality rate was 12.3% and there was no difference in 30-day mortality according to serotypes (log rank test *P*=0.39). In logistic regression analysis, high SOFA score was significantly associated with 30-day mortality (odd ratio 1.28, 95% C.I. 1.28-2.53, *P*< 0.01). All isolated GBS were susceptible to penicillin and resistances were identified against clindamycin, erythromycin and levofloxacin as 30%, 26.9%, 24.6% of the isolates, respectively.

**Conclusion:**

We found the most common capsular serotype was VIII (31.5%) in Korea and the proportion of type VIII increasing over time. It is necessary to consider GBS vaccine in adults with underlying disease and its composition for serotype VIII according to epidemiologic characteristics.

**Disclosures:**

**All Authors**: No reported disclosures

